# Optimal temperature and thermal tolerance of postlarvae of the freshwater prawn *Cryphiops (Cryphiops) caementarius* acclimated to different temperatures

**DOI:** 10.1016/j.heliyon.2024.e25850

**Published:** 2024-02-10

**Authors:** Karla Ferrer-Chujutalli, José Sernaqué-Jacinto, Walter Reyes-Avalos

**Affiliations:** a Escuela Profesional de Biología en Acuicultura, Universidad Nacional del Santa, Ancash, 02712, Perú; b Laboratorio de Acuicultura Ornamental, Departamento Académico de Biología, Microbiología y Biotecnología, Universidad Nacional del Santa, Ancash, 02712, Perú

**Keywords:** Growth parameters, Acclimation capacity, Thermal safety margin, Thermal tolerance interval, Acclimation rate response, Thermal indicators

## Abstract

In this study, the optimum temperature and thermal tolerance of postlarvae of the commercially important freshwater prawn *Cryphiops (Cryphiops) caementarius* were determined after acclimation to six different rearing temperatures (19 °C, 22 °C, 24 °C, 26 °C, 28 °C, and 30 °C) during a 45 day-culture period. Best growth parameter values were obtained within the temperature range of 24 °C to 28 °C, where the optimum temperature for growth was found to be at 26 °C (weight gain 81.70%; specific growth rate 1.33 %/day) but had not significant effect (*p* > 0.05) on survival (64%–71%) of postlarvae. Increasing the acclimation temperature significantly (*p* < 0.05) increased both the critical thermal maximum (CTMax: from 33.82 °C to 38.48 °C) and minimum (CTMin: from 9.27 °C to 14.58 °C). The thermal tolerance interval increased (*p* < 0.05) from 24.55 °C to 25.48 °C in postlarvae acclimated at 28 °C but decreased (*p* < 0.05) to 23.90 °C in those acclimated at 30 °C. The acclimation response rate was lower for CTMax and higher for CTMin. The current (12.48 °C) and future (9.48 °C) thermal safety margins were like those reported for other tropical crustaceans. A thermal tolerance polygon over the range of 19–30 °C resulted in a calculated area of 242.25 °C^2^. The presented results can be used for aquaculture activities and also to help to protect this species against expected climate warming impacts.

## Highlights

• The optimal temperature for growth of *C. (C.) caementarius* postlarvae is 26 °C.
• Acclimation at 19-30 °C increases critical temperatures in *C. (C.) caementarius* postlarvae.
• The thermal tolerance polygon over the range of 19–30 °C was 242.25°C^2^.
• Postlarvae of *C. (C.) caementarius* adapt to thermal variations in water.

## 1 Introduction

Climate change alters ecosystems and their diversity, forcing all organisms to adapt, migrate, or perish; and the survival rate of organisms will depend on their life history, dispersal in relation to habitat fragmentation, and the rate of environmental change [1]. Freshwater habitats are less stable to temperature shifts because they face daily and seasonal variability [2]. These conditions affect ectothermic organisms such as crustaceans, which may suffer alterations in energetic costs and biochemical and behavioral changes [3,4].

The optimal temperature is characterized by the functional optimum of the aerobic range between critical temperatures, where the organism's performance is at its greatest [5]. The optimum growth temperature for postlarvae and juveniles of tropical crustaceans such as *Macrobrachium tenellum*, and *Cherax quadricarinatus* is between 29 °C and 31 °C [6,7]. On the other hand, in crustaceans from temperate climates such as *Palaemonetes argentinus* and *M. borellii*, the optimum temperature for growth lies between 20 °C and 25 °C [8].

Thermal tolerance is determined by critical thermal parameters, with the aerobic range decreasing and affecting the survival of the organism [9]. In crustaceans, acclimated temperature affects the response to thermal tolerance by increasing the critical thermal maximum (CTMax) and critical thermal minimum (CTMin) before equilibrium is lost and death occurs [10]. This has been observed in adults *Procambarus clarkii* [11], *M. acanthurus* [12], juvenile *C. quadricarinatus* [13], *Samastacus spinifrons* [14], and *M. tenellum* [6], although each species has its own thermal responses. In this way, the information from CTMax and CTMin allows for the estimating of the Acclimation Rate Response (ARR), Acclimation Capacity (AC), Thermal Tolerance Interval (TTI), and current (TSM), and future (FTSM) Thermal Safety Margin, which is useful for understanding the thermal ecology of organisms in the face of climate warming, and thus are valuable reference values for studies of aquatic species [3,15,16]. Likewise, temperature tolerance polygons provide information on the ability of each species to mitigate the effects of climate change [17].

*Cryphiops (Cryphiops) caementarius* (formerly known as *Macrobrachium caementarius*, see Mantelatto et al. [18]) is a temperate climate prawn species of high commercial value that inhabits the western coast rivers of Peru and Chile [19]. The highest population densities of *C. (C.) caementarius* are found in rivers located in temperate and arid regions of the southern Peruvian coast, whose water temperatures vary between 16 °C and 29 °C throughout the year [20,21]. However, it has been reported that Chilean populations of this species tolerate seasonal thermal oscillations. For example, *C. (C.) caementarius* populations from Limarí River withstand thermal oscillations from 15 °C to 24 °C in summer and 12 °C–15 °C in winter, while populations from Choapa River inhabit waters with temperatures ranging from 10.5 °C to 15 °C in the winter and 14.5 °C–26.6 °C in summer [22,23]. The wide variation in habitat temperature within the range of latitudinal distribution is already an indicator of this species’ thermal tolerance since its juveniles have a thermal range adjusted to extremes of 8 °C and 28 °C [24].

Information on the optimum temperature and thermal tolerance is not only necessary to determine the best cultivation sites and proper aquaculture management but also to know species’ thermal response to climate change. Even though the thermal preference of *C. (C.) caementarius* postlarvae was previously determined [25], the optimum temperature for their growth is still unknown and no studies have been carried out to determine their thermal tolerance. Therefore, this study aimed to determine the optimum temperature and thermal tolerance of postlarvae of the freshwater prawn *C. (C.) caementarius* acclimated to different temperatures.

## 2 Materials and methods

### 2.1 Ethical statement

The experiments conducted in this study were performed under the Peruvian Law of Animal Protection and Welfare (Law 30,407, Animal Protection and Welfare Law).

### 2.2 Capture, transport, and acclimation of the freshwater prawns

*Cryphiops (C.) caementarius* prawn postlarvae were captured near (≈100 m) the mouth of Mala River (12°40′30″ S, 76°39′30″ W) (Lima, Peru) in September 2021, where the water temperature was 19.2 °C. The postlarvae were conditioned and transported in plastic bags with 10 L of water from the same river at a density of 200 postlarvae/L. The land transport from the collection site to the laboratory lasted 16 h. Acclimation was carried out for 20 days in 55-L aquariums with fresh water at room temperature (19.3 ± 0.3 °C). Postlarvae were fed a balanced diet (30% crude protein). Aquariums were cleaned and water changes (20%) were performed three times a week.

### 2.3 Selection and growing conditions

A total of 540 postlarvae (1.2 ± 0.2 cm total length and 0.53 ± 0.01 g total weight) were selected and cultured for 45 days in 18 aquariums (0.30 m long, 0.20 m wide and 0.20 m high, with 0.06 m^2^ and 10 L), with brackish water at 12‰ and 500 postlarvae/m^2^ [26]. The water temperature of the aquariums in each treatment (19 °C, 22 °C, 24 °C, 26 °C, 28 °C, and 30 °C) was increased at a rate of 1 °C/h using thermoregulators (100 W; ±0.5 °C). Each treatment was carried out in triplicate. Aeration (1.5 L/min) was kept constant to allow oxygen diffusion and to avoid thermal stratification. Balanced feed (30% crude protein) formulated for adults of *C. (C.) caementarius* prawns was used [27]. The daily ratio (08:00 and 18:00 h) was 10% of the wet biomass, which was readjusted every 15 days after each sampling.

### 2.4 Water quality

The water temperature of the aquarium was determined daily with a digital thermometer (±0.01 °C). The chemical parameters of the aquarium water were monitored weekly, and dissolved oxygen was determined with a digital oximeter (±0.01 mg/L), pH with a digital pH meter (±0.01 units), and total ammonium and nitrite with colorimetric kits (±0.25 mg/L).

### 2.5 Growth and survival parameters

Postlarvae weight was determined using the volumetric method [26] and weight gained (WG), specific growth rate (SGR), and survival (S) were calculated using the following formulae:

WG (%) = (Final weight - Initial weight/Initial weight) × 100.

SGR (%/day) = [(ln Final weight) - (ln Initial weight)/Duration of culture] × 100.

S (%) = (Final number of prawn / Initial number of prawn) × 100

### 2.6 Thermal experimental system

The water heating system consisted of a 10 L container connected to a thermoregulator and the cooling system was a 4 L container with frozen hydrogel bags. The thermal variation of the system was ±0.1 °C/min [28]. The heating and cooling rate of the water used to calculate the CTMax and CTMin was as fast as 1.1 ± 0.1 °C/min, similar to that suggested for marine [28] and freshwater crustaceans [29,30].

### 2.7 Critical thermal limits

The critical thermal limits were determined in the postlarvae that came from the optimal temperature experiment. The estimation of CTMax and CTMin was performed with the critical thermal method using six postlarvae randomly collected from thermal treatment (two per replicate). The postlarvae were exposed to a linear decrease or increase of temperature. Each animal was used once only. To avoid stress, each prawn was placed in a flexible plastic cup (250 mL) with water at the same acclimation conditions (temperature, salinity, and aeration) and maintained for 2 min. The endpoint was considered when temperature caused total disorientation and locomotor disorganization of the postlarvae [31], and water temperature was immediately reduced or increased (2 °C/h) by adding water until their respective acclimation temperatures were reached. Then, the survival of the prawns was evaluated for one week.

### 2.8 Thermal indicators

The ARR of postlarvae was calculated according to Claussen [32]. A TTI was estimated according to Syafaat et al. [28], while the coefficient of variation (CV) from CTMax and CTMin, and CA according to Madeira et al. [15]. The TSM was estimated for each acclimation temperature, and the FTSM for the year 2100 was calculated according to Vinagre et al. [16]. The maximum annual mean temperature at the mouth of the Mala River was 26 °C [33].

ARR=ΔCTMaxorΔCTMin/Δacclimationtemperature

TTI(°C)=CTMax–CTMin

CV(%)=(Standarddeviation/Mean)×100

AC(°C)=CTat30°C–CTat19°C

TSM(°C)=CTMax–Maximumhabitattemperature

FTSM(°C)=CTMax–(Maximumhabitattemperature+3°C)

### 2.9 Thermal tolerance polygon

The thermal tolerance polygon and the estimation of the area (°C^2^) were obtained according to Bennett and Beitinger [34], and the intrinsic tolerance and upper and lower acquired tolerance areas were determined according to Eme and Bennett [17].

### 2.10 Statistical analysis

Growth, survival, and thermal tolerance data were analyzed using the completely randomized statistical design. The normality of the data was analyzed with the Shapiro-Wilk test, and equality of variance by Levene's test. A linear regression was then performed correlating acclimation temperature against CTMax or CTMin, the significance level was set at *p* < 0.01. Differences between treatments were determined by one-way analysis of variance (ANOVA) and Duncan's post hoc test. Animal mass may have a potential impact on heat tolerance; therefore, a one-way analysis of covariance (ANCOVA) was performed to test the effect of acclimation temperature as a fixed factor and total weight as a covariate, on the CTMax and CTMin. The differences were considered statistically significant when *p* < 0.05. Data were recorded as mean values with a standard deviation (SD). Statistical tests were performed using SPSS version 25.

## 3 Results

### 3.1 Optimum temperature

Growth parameter values determined for *C. (C.) caementarius* postlarvae at various acclimation temperatures are illustrated in Table 1. The highest growth parameters in weight of the postlarvae were obtained at temperatures up to 28 °C, with the greatest growth trend achieved at 26 °C. On the other hand, the lowest growth was obtained at 30 °C. The ANOVA analysis results showed that the acclimation temperature did not affect survival (*p* > 0.05). Water quality parameters during the 45 days of culture were similar (*p* > 0.05) among the six temperature treatments, where oxygen ranged from 5.12 to 6.68 mg/L, pH from 7.15 to 7.42, ammonium from 0.32 to 0.43 mg/L, and nitrite from 1.25 to 2.75 mg/L.

**Table 1:** Growth parameters (mean ± standard deviation) of *C. (C.) caementarius* postlarvae cultured for 45 days in brackish water (12‰) at different temperatures.

Parameters	Temperature (°C)					
	19	22	24	26	28	30
TW (g)	0.88 ± 0.07^ab^	0.86 ± 0.05^bc^	0.92 ± 0.05^ab^	0.96 ± 0.05^a^	0.88 ± 0.02^ab^	0.78 ± 0.05^c^
WG (%)	66.10 ± 12.48^ab^	63.08 ± 8.73^bc^	74.40 ± 8.79^ab^	81.70 ± 9.82^a^	66.73 ± 3.68^ab^	47.11 ± 9.42^c^
SGR (%/day)	1.21 ± 0.12^ab^	1.09 ± 0.12^bc^	1.23 ± 0.11^ab^	1.33 ± 0.12^a^	1.14 ± 0.05^ab^	0.87 ± 0.13^c^
S (%)	71.11 ± 3.85^a^	67.78 ± 3.85^a^	70.00 ± 3.33^a^	71.11 ± 7.70^a^	64.44 ± 5.09^a^	70.00 ± 3.30^a^

TW: Total weight. WG: Weight gain. SGR: Specific growth rate. S: Survival. Data with different superscripts in the same row indicate significant differences (*p* < 0.05).

### 3.2 Thermal tolerance and thermal indicators

Prawn weight had no effect (ANCOVA, *p* < 0.05) on CTMax (Supplementary Table S1) and CTMin (Supplementary Table S2). A direct relationship between acclimation temperature with CTMax and with CTMin was observed. Thermal tolerance for CTMax significantly (*p* < 0.05) increased from 34 °C ± 0.26–38.48 °C ± 0.27 as acclimation temperature increased from 22 °C to 30 °C, whereas CTMin values increased from 9.27 °C ± 0.54–14.58 °C ± 0.95 as acclimation temperatures increased from 19 °C to 30 °C (Table 2). TTI values increased among acclimated temperatures of 22 °C–28 °C but decreased at 30 °C. The calculated AC was 4.66 °C and 5.31 °C for CTMax for CTMin, respectively. The TSM and FTSM were calculated at 12.48 °C at 9.48 °C, respectively.

**Table 2:** Thermal tolerance and thermal tolerance interval (TTI) of *C. (C.) caementarius* postlarvae cultured for 45 days in brackish water (12‰) at different temperatures (mean ± standard deviation). The corresponding coefficients of variation are shown in the brackets.

Acclimation temperature (°C)	Thermal tolerance		TTI (°C)
	CTMax (°C)	CTMin (°C)	
19	33.82 ± 0.27^a^ (0.80%)	9.27 ± 0.54^a^ (5.31%)	24.55
22	34.00 ± 0.26^a^ (0.77%)	10.27 ± 0.22^b^ (1.92%)	23.73
24	34.85 ± 0.45^b^ (1.28%)	10.53 ± 0.21^b^ (1.79%)	24.32
26	36.15 ± 0.38^c^ (1.06%)	10.68 ± 0.37^b^ (3.12%)	25.47
28	37.12 ± 0.23^d^ (0.62%)	11.63 ± 0.52^c^ (4.11%)	25.48
30	38.48 ± 0.27^e^ (0.71%)	14.58 ± 0.95^d^ (5.92%)	23.90

Each value is a mean ± SD (n = 6 postlarvae). Data with different superscripts in the same column indicate significant differences (*p* < 0.05).

The ARR increased for CTMax and CTMin with acclimated temperatures, with averages of 0.46 and 0.50, respectively. The highest ARR CTMax in relation to ARR CTMin was obtained in the temperature ranges of 22–24 °C and 24–26 °C. In contrast, the highest ARR CTMin in relation to the ARR CTMax was in the ranges of 19–22 °C and 28–30 °C (Table 3).

**Table 3:** The acclimation rate response (ARR) of *C. (C.) caementarius* postlarvae cultured for 45 days in brackish water (12‰) at different temperatures (mean ± standard deviation).

Acclimation temperature (°C)	ARR	
	CTMax	CTMin
19–22	0.06	0.33
22–24	0.43	0.13
24–26	0.65	0.08
26–28	0.48	0.48
28–30	0.68	1.48
Mean	0.46 ± 0.25	0.50 ± 0.57

Data with different superscripts in the same column indicate significant differences (*p* < 0.05).

### 3.3 Thermal tolerance polygon

**Fig. 1:**
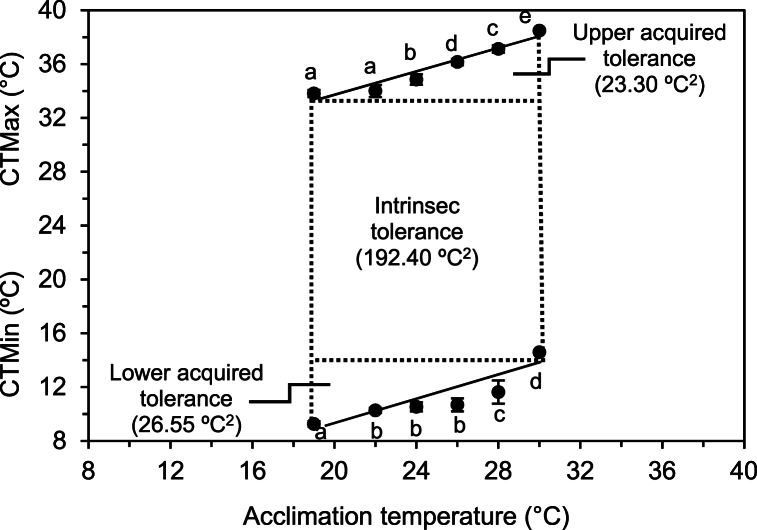
Thermal tolerance polygon of *C*. *(C) caementarius* postlarvae at different temperatures (mean ± standard deviation). Different letters in the continuous line straight indicate significant differences (*p* < 0.01).

The acclimated temperature significantly (*p* < 0.01) affected CTMax and CTMin. Differences in acclimated temperature explained 93.2% of the variance of CTMax and 78.2% of CTMin. The area of the total thermal tolerance polygon delimited by the CTMin and CTMax for each acclimation temperature (from 19 °C to 30 °C) exposed to *C. (C.) caementarius* postlarvae resulted in a calculated area of 242.25 °C^2^ (Fig. 1). There were high and positive correlations between acclimation temperatures and CTMax (Y = 0.44X + 24.71; r = 0.96; R^2^ = 0.93; *p* < 0.01) and CTMin (Y = 0.40X + 1.11; r = 0.88; R^2^ = 0.78; *p* < 0.01). The intrinsic thermal tolerance was 192.40 °C^2^, representing 79.42% of the area of the thermal tolerance polygon. The sum of the two acquired tolerance areas was 49.85 °C^2^ (20.58% of the total area). The upper and lower acquired tolerance area was 23.30 °C^2^ (9.62%) and 26.55 °C^2^ (10.96%), respectively (Fig. 1).

## 4 Discussion

### 4.1 Optimal temperature

The results of this study showed that postlarvae of *C. (C.) caementarius* can grow and survive for 45 days in a wide acclimating temperature range (19 °C to 30 °C). However, higher growth was achieved between 24 °C and 28 °C with the highest SGR and WG obtained at 26 °C, which can be considered optimal for effective aquaculture production. Similar results were reported in juveniles of other prawn species. For example, in *Palaemon elegans* growth rates are highest between 21 °C and 27 °C, with the optimum being 24 °C [35], while in *C. quadricarinatus*, it is desirable at 28 °C, although, physiological processes are more efficient at 25 °C [7]. In juveniles of *M. borellii* and *P. argentinus*, culture is optimal between 20 °C and 25 °C to increase growth rate and molt frequency [8]. In juveniles of *M. occidentale*, the optimal temperature is between 25 °C and 28 °C, but growth is rapid at the latter temperature [29]. The temperature for optimal growth (26 °C) of *C. (C.) caementarius* postlarvae revealed in this study is higher than its preferred temperature (24.5 °C) [25]. This finding is in agreement with the results of Westhoff and Rosenberger [30], who reported that in freshwater crustaceans the temperature for optimal growth always exceeds the thermal preference. A possible reason for the observed higher optimal temperature for growth may be due to that species from low-productivity waters optimize growth efficiency rather than growth rate [36].

In this study, *C. (C.) caementarius* postlarvae cultured at 30 °C showed less weight, indicating that this temperature is detrimental for the species. Juveniles of this species experiment stress at 30 °C because energy demands exceed individual reserves [24]. Similar results were obtained with juveniles of *M. borellii* and *P. argentinus* grown at 30 °C, where there was a reduction in growth due to energy shortage, even though they were fed in excess [8]. It has also been reported that in *Aegla longirostri*, high temperatures produce biochemical changes in muscle and hepatopancreas [4].

In the current study, culture water temperature (19 °C–30 °C) did not affect survival (64%–71%) of *C. (C.) caementarius* during the 45-day culture period. However, in *M. borellii* and *P. argentinus*, high survival (95%) is maintained when cultured at temperatures between 20 °C and 25 °C but decreased at 30 °C (76% and 48% in each species, respectively) [8]. In *C. quadricarinatus*, the highest survival (83%) is achieved only at 25 °C, where physiological processes are more efficient [7]. Similarly, high survival rates (92%) in *M. occidentale* were obtained at 25 °C [29]. A possible reason why survival rates were not drastically affected by our temperature treatments may be due to the use of brackish water (12‰ salinity), which has been shown to decrease cannibalism in prawn species [26] and to decrease nitrite toxicity [37] due to the presence of chloride ions in the water [38]. Therefore, the high nitrite content (1.25–2.75 mg/L) did not have a severe effect on the postlarvae survival, even with the rise in temperature. However, it has been suggested that water for freshwater prawn culture should contain less than 1.0 mg/L nitrite [39].

### 4.2 Thermal tolerance

Several potentially complicating factors, such as thermal history, acclimated temperature, and rate of temperature change, may affect the resultant target water temperature thresholds [40]. In our research, these criteria were covered, and for the thermal history, we used only specimens with a total length of 1.2 cm (corresponding to the first postlarvae stage, Morales et al. [41]), which were kept for 45 days at the different acclimated temperatures. We also considered the period of larval development (77 days) to postlarvae [42], the time that postlarvae stay in estuaries (4 days) before migrating [43], and the days of acclimation (20 days) in the laboratory. The recent thermal history alters thermal tolerance and survival in crustaceans [44]. Besides, a long duration of the thermal acclimated period may lead to more accurate predictions of future biodiversity patterns than those of short duration [3].

In crustaceans, acclimated temperature affects the response to thermal tolerance [10]. In this work, the acclimation temperature treatments (19 °C to 30 °C) caused an increase of both CTMax (from 33.82 °C to 38.48 °C) and CTMin (from 9.27 °C to 14.58 °C) of *C. (C.) caementarius* postlarvae. In *M. rosenbergii* postlarvae, CTMax increases from 36.3 °C to 41.6 °C and CTMin from 10.0 °C to 16.8 °C, at acclimated temperatures between 20 °C and 32 °C, respectively [45]. In juveniles of *C. quadricarinatus*, CTMax increases from 36 °C to 42 °C in acclimated temperatures between 20 °C and 32 °C [13]. Furthermore, our results showed that there was relatively less variation of CTMax (0.62%–1.28%) compared to CTMin (1.79%–5.92%), indicating that *C. (C.) caementarius* postlarvae are more sensitive to heat stress than to cold stress.

The observed CTMax (38.48 °C) and CTMin (9.27 °C) values of *C. (C.) caementarius* indicate that this species has high thermal tolerance. Crustaceans that spend part of their life cycle in estuaries must have adaptive mechanisms to cope with thermal variations [46]. Among those mechanisms, heat shock proteins that are produced during heat or cold stress [47] and certain ions that are released to reduce heat loss [48] are expressed in embryos, larvae [49], juveniles [50], and adult [51] crustaceans. A previous study [52] reported that newly hatched larvae of *C. (C.) caementarius* without thermal acclimation altered their swimming behavior in response to the abrupt increase (22 °C–32 °C) or decrease (22 °C–10 °C) in water temperature, allowing them to survive. According to this research, *C. (C.) caementarius* postlarvae are born with the ability to withstand extreme thermal variations, thus ensuring the survival of offspring in places with highly fluctuating environmental conditions.

### 4.3 Acclimation rate response

In *C. (C.) caementarius* postlarvae, the acclimated temperatures caused greater amplitude in ARR variation, from 0.06 to 0.68 for CTMax and from 0.33 to 1.48 for CTMin. The observed ARR variation is likely to be a consequence of the arid and temperate climates of the environment [21] where this prawn species inhabits, suggesting greater plasticity in physiological function in response to temperature changes. The higher amplitude of the ARR indicates high thermal tolerance after acclimation to elevated temperatures [53], as the ARR describes the short- and long-term response rates to environmental changes [29] and represents the window within the upper half of the species' thermal niche [3].

Our results on the ARR of *C. (C.) caementarius* also revealed that the highest plasticity to heat was observed only at acclimated temperatures of 22–24 °C and 24–26 °C where the ARR of CTMax was higher than the ARR of CTMin. In contrast, the highest plasticity to cold occurred at temperatures greater than 26 °C and less than 22 °C, where the ARR of CTMin was higher than the ARR of CTMax. These results suggest that *C. (C.) caementarius* postlarvae have higher cold adaptability but low heat tolerance, which could be explained by the high value (1.48) for CTMin that has not been yet reported in other crustaceans, but an ARR of 1 is known to indicate complete compensation for acclimation temperature [54]. This thermal plasticity of postlarvae is likely a characteristic of this ontogenetic stage inhabiting estuarine zones, where environmental conditions are highly changeable.

### 4.4 Thermal tolerance interval

The TTI values of *C. (C.) caementarius* postlarvae obtained herein showed an increment from 23.73 °C to 25.48 °C with acclimated temperatures from 22 °C to 28 °C, respectively. This confirms previous reports on postlarvae of the same species whose thermal optimum zone is in the range of 23.2 °C to 25.8 °C [25]. The TTI values obtained in this work are also similar to those of tropical prawns such as *M. rosenbergii* (24.8 °C to 27.3 °C) [45], although it was higher than that of *M. acanthurus* (22.9 °C to 23.6 °C) [12]. A high ITT represents a physiological change that allows organisms enduring fluctuating temperatures to survive at higher temperatures than those acclimated to constant temperature [55].

In contrast, the decrease in TTI to 23.90 °C with the acclimated temperature of 30 °C is a response of *C. (C.) caementarius* postlarvae to chronic thermal stress that also affects growth, which is likely due to some alteration of metabolism due to the 45-day culture period. In ectotherms, the increase in temperature involves increased energy expenditure or additional costs associated with the adjustment of physiological pathways [3]. In addition, reduced TTI is related to ecological advantages associated with habitats that have optimal temperatures for growth [56], which in *C. (C.) caementarius* postlarvae was found to be 26 °C, and as above mentioned, was higher than its preferred temperature of 24.5 °C [25].

### 4.5 Acclimation capacity

The gradual increase in water temperature due to global climate change has been estimated in the range of 2.4 °C to 4.5 °C by the year 2100 [57]. In this extended period, natural acclimation to withstand extreme temperatures could occur, mainly in fish and crustaceans from temperate climates, which have a higher tolerance to temperature increases than those from tropical areas [58]. The AC is the change of a physiological parameter for the organism to adapt to new environmental conditions [15]. In this sense, there is a direct relationship between critical temperatures with acclimated temperatures in freshwater crustaceans and their AC varies from 2 °C to 6 °C, as in *M. tenellum* [59], *M. rosenbergii* [45], *M. occidentale* [29], among many others. In this study, the AC of *C. (C.) caementarius* postlarvae increased with acclimated temperature by 4.66 °C for CTMax and by 5.31 °C for CTMin, which suggests that this species has the potential to colonize other environments in the face of future climate change.

### 4.6 Thermal safety margin

TSM is the comparison of the maximum temperature that an organism can withstand with the maximum temperature of the environment [15]. The *C. (C.) caementarius* postlarvae has a TSM of 12.48 °C and an FTSM of 9.48 °C, similar to that reported for coastal marine crustaceans in temperate and tropical climates, which have broad TSM of 8 °C–13 °C and FTSM of 5 °C–10 °C [16]. Ectotherms that have broad TSM are known to live in environments that, on average, are greater than optimal, and as temperature increases, they experience initial increases in growth rates and organism performance [60]. This means that if there are abrupt increases in habitat water temperature mainly in the austral summer months and in the context of future climate change, *C. (C.) caementarius* postlarvae can withstand temperatures up to 9.48 °C higher than the ambient average before reaching fatal critical levels. However, TSM from other ontogenetic stages of *C. (C.) caementarius* are yet to be determined.

### 4.7 Thermal tolerance polygon

In the present work, the area of the thermal tolerance polygon of *C. (C.) caementarius* acclimated to rearing conditions between 19 °C and 30 °C was 242.25 °C^2^, whose central position (Fig. 1) is characteristic of eurytherms [61]. A similar thermal tolerance area (225 °C^2^) was obtained in juveniles of *C. quadricarinatus* acclimated between 20 °C and 32 °C [13] and in *M. rosenbergii* (255 °C^2^) acclimated between 25 °C and 35 °C [62]; although it was lower than *M. acanthurus* (644 °C^2^) acclimated between 20 °C and 32 °C [12].

The intrinsic thermal area of the total thermal tolerance polygon of *C. (C.) caementarius* was 192.40 °C^2^ (Fig. 1), which represented 79.42% of the total thermal niche, characteristic of organisms with moderately large tolerance, but at the same time preserving the ability to acquire additional tolerance if needed [61]. Furthermore, the midpoint within this intrinsic zone corresponds to the thermal preference of *C. (C.) caementarius* (24.5 °C) [25] and to the optimal temperature range (24 °C–28 °C), although those indicators can move within the limits of this zone. Regarding the acquired thermal tolerance, which is acclimation-dependent [63], the area of the lower acquired zone (26.55 °C^2^) was slightly larger than the upper acquired zone (23.30 °C^2^), indicating a slight tolerance to cold rather than high temperatures. This thermogenic behavior was also observed in *C. (C.) caementarius* larvae, whose exposure to low temperatures is not detrimental to their survival and vitality, unlike high temperatures [52].

## 5 Conclusions

This study determined for the first time that the optimum temperature for the growth of postlarvae of the freshwater prawn *C. (C.) caementarius* is 26 °C. Regarding the thermal tolerance, our results demonstrated that increasing the acclimated temperatures also increases the CTMax and CTMin, and the total thermal tolerance polygon area was 242.25 °C^2^, characteristic of eurytherm animals. In addition, the thermal indicators demonstrated that the postlarvae of this prawn species have the capacity to adapt to a wide range of water thermal variations, which can enable them to cope with future climate change. The results obtained in this research can serve as baseline data for improving aquaculture production of this species and for further thermal ecology studies aimed to help to predict the responses of the different ontogenetic stages of this prawn species.

## Data statement

Data will be made available on request.

## Additional information

No additional information is available for this paper.

## Funding source

This research did not receive any specific grant from funding agencies in the public, commercial, or not-for-profit sectors.

## CRediT authorship contribution statement

**Karla Ferrer-Chujutalli:** Writing – original draft, Writing – review & editing, Methodology, Formal analysis, Conceptualization. **José Sernaqué-Jacinto:** Writing – original draft, Methodology, Formal analysis, Conceptualization. **Walter Reyes-Avalos:** Writing – original draft, Writing – review & editing, Validation, Supervision, Conceptualization.

## Declaration of competing interest

The authors declare that they have no financial interests or personal relationships that could have influenced this work.
