# Supplementary material for: Optimal temperature and thermal tolerance of postlarvae of the freshwater prawn Cryphiops (Cryphiops) caementarius acclimated to different temperatures
Source: Heliyon. 2024 Feb 10;10(5):e25850. doi: 10.1016/j.heliyon.2024.e25850 (PMC10907542; doi:10.1016/j.heliyon.2024.e25850)
Supplement: Multimedia component 1 [file mmc1.docx]

**Supplementary material**

**Table S1**: ANCOVA results on the interaction effect of acclimation temperature and total weight on the CTMax.

| Dependent Variable: CTMax | | |  |  |  |  |
| --- | --- | --- | --- | --- | --- | --- |
|  | **Tests of Between-Subjects Effects** | | | | | |
| Origen | Type III Sum of  Squares | df | Mean  Square | F | Sig. | Partial Eta  Squared |
| Corrected Model  Intercept  Acclimation temperature  Total weight  Acclim. Temp * Total weight  Error  Total  Corrected Total | 51,933^a^  30,426  ,404  ,175  ,248  0,370  23071,730  52,303 | 11  1  5  1  5  6  18  17 | 4,721  30,426  ,081  ,175  ,050  0,062 | 76,549  493,338  1,311  2,840  ,806 | ,000  ,000  ,371  ,143  ,585 | ,993  ,988  ,522  ,321  ,402 |
| a. R Squared = ,993 (Adjusted R Squared = ,980) | | |  |  |  |  |

**Table S2**: ANCOVA results on the interaction effect of acclimation temperature and total weight on the CTMin.

| Dependent Variable: CTMin | | |  |  |  |  |
| --- | --- | --- | --- | --- | --- | --- |
|  | **Tests of Between-Subjects Effects** | | | | | |
| Origen | Type III Sum of  Squares | df | Mean  Square | F | Sig. | Partial Eta  Squared |
| Corrected Model  Intercept  Acclimation temperature  Total weight  Acclim. temp * Total weight  Error  Total  Corrected Total | 51,306^a^  1,891  ,644  ,043  ,519  ,699  2303,210  52,005 | 11  1  5  1  5  6  18  17 | 4,664  1,891  ,129  ,043  ,104  ,116 | 40,042  16,233  1,106  ,373  ,891 | ,000  ,007  ,445  ,564  ,541 | ,987  ,730  ,480  ,059  ,426 |
| a. R Squared = ,987 (Adjusted R Squared = ,962) | | |  |  |  |  |
